# Nonlinear Secret Image Sharing Scheme

**DOI:** 10.1155/2014/418090

**Published:** 2014-07-21

**Authors:** Sang-Ho Shin, Gil-Je Lee, Kee-Young Yoo

**Affiliations:** School of Computer Science and Engineering, Kyungpook National University, 80 Daehakro, Bukgu, Daegu 702-701, Republic of Korea

## Abstract

Over the past decade, most of secret image sharing schemes have been proposed by using Shamir's technique. It is based on a linear combination polynomial arithmetic. Although Shamir's technique based secret image sharing schemes are efficient and scalable for various environments, there exists a security threat such as Tompa-Woll attack. Renvall and Ding proposed a new secret sharing technique based on nonlinear combination polynomial arithmetic in order to solve this threat. It is hard to apply to the secret image sharing. In this paper, we propose a (*t*, *n*)-threshold nonlinear secret image sharing scheme with steganography concept. In order to achieve a suitable and secure secret image sharing scheme, we adapt a modified LSB embedding technique with XOR Boolean algebra operation, define a new variable *m*, and change a range of prime *p* in sharing procedure. In order to evaluate efficiency and security of proposed scheme, we use the embedding capacity and PSNR. As a result of it, average value of PSNR and embedding capacity are 44.78 (dB) and 1.74*t*⌈log_2_⁡*m*⌉ bit-per-pixel (bpp), respectively.

## 1. Introduction

In a security system, there is a maintenance tool which must be checked every day. In order to check it, someone must have access to this system. Three senior administrators are engaged, but they do not trust the combination to any individual administrator. Hence, we would like to design a system whereby any two of three administrators can gain access to this system, but an individual administrator cannot do so. In order to design this system, we adapt a concept of secret sharing. A secret sharing is technique for distributing a secret amongst a group of honest participants, and each secret piece is allocated for each participant after the secret is divided into several pieces. This secret can be reconstructed only when a sufficient number, of possibly different types of shares are combined together; an individual share is no use on its own [1–3]. Blakley [4] and Shamir [5] have proposed a concept of secret sharing for the first time. It is that the secret is divided into *n* shares for *n* participants, and *t* is used as a threshold value (*t* ≤ *n*). It was called a (*t*, *n*)-threshold technique and it means that at least *t* participants of *n* participants should be gathered.

With the development of computing and network technologies, in the meantime, multimedia data such as image, audio, and video files have transmitted over the Internet, actively. As a result, multimedia security has emerged as an important issue [6–14]. In 2002, Thien and Lin [15] have proposed a (*t*, *n*)-threshold secret image sharing scheme for the first time. The secret image can be shared by several shadow images so the size of each shadow image is only 1/*t* of that of the secret image for convenient hiding, storage, and transmission in their scheme. Lin and Tsai [16] have proposed a secret image sharing with steganography concept based on the Shamir's (*t*, *n*)-threshold scheme. By using the parity bit check method, they claimed that their scheme can prevent from incidentally bringing an erroneous shadow image or intentionally providing a false image to achieve the authentication goal. They also presented another user-friendly image sharing such that shadow images look like natural images [17]. Recently, Lin and Chan [18] proposed a reversible secret image sharing scheme in 2010. They have achieved a low distortion and high embedding capacity. Additionally, it can reconstruct the secret and cover images, completely.

As mentioned above, most of secret image sharing schemes are based on Shamir's (*t*, *n*)-threshold. It has utilized a linear combination polynomial arithmetic in sharing procedure. A configuration of the linear combination polynomial with (*t*, *n*) is as follows:

(1) $$\begin{array}{c} f\left(x\right)=a_{0}+a_{1}x+a_{2}x^{2}+\cdots +a_{t-1}x^{t-1}. \end{array}$$

In sharing procedure for *n* participants, an arbitrary share *k*_*i*_ (1 ≤ *i* ≤ *n*) is computed by above function *f*(*x*). Each share *k*_*i*_ is distributed to participant *i*. In order to reconstruct the secret, we need *t* pairs of (*i*, *k*_*i*_). In this procedure, an arbitrary participant can submit a false share and only he will be able to obtain the correct secret while leaving the others with the incorrect secret. It is called a Tompa-Woll attack [19, 20]. This attack is caused by the linear property. Renvall and Ding [21] have proposed a new secret sharing scheme based on a nonlinear combination polynomial arithmetic in order to solve this problem. The nonlinear combination arithmetic indicates an inner product for any arbitrary matrix [21]. However, it is hard to apply to the secret image sharing.

In this paper, we propose a nonlinear secret image sharing scheme with steganography concept. Although the proposed scheme is based on Renvall and Ding's sharing and reconstruction methods [21], we adapt several new techniques in order to achieve a suitable and secure secret image sharing scheme. In sharing procedure, we define a new variable *m* and change a range of prime *p* in order to attain the prevention of overflow (or underflow) and reinforce the security. Also, we propose a modified LSB embedding technique with XOR Boolean algebra operation in order to get the high embedding capacity. In order to evaluate efficiency and security of proposed scheme, we use the embedding capacity and PSNR. As the experimental results, we analyze the efficiency and security between proposed and previous techniques.

This paper is organized as follows.  Section 2 introduces Shamir's and Renvall and Ding's secret sharing scheme. Considerations and algorithm of proposed scheme are discussed in Section 3.  Section 4 presents the experimental results. Lastly, Section 5 gives the conclusions.

## 2. Preliminaries

In this section, Shamir's (*t*, *n*)-threshold secret sharing and Renvall and Ding's nonlinear secret sharing are introduced.

### 2.1. Shamir's (*t*,  *n*)-Threshold Secret Sharing

In 1979, Shamir has proposed a secret sharing scheme for the first time [5]. It is based on (*t*, *n*)-threshold which is defined as follows [2].

**Definition 1 .**

Let *t*, *n* be positive integers and *t* ≤ *n*. A (*t*, *n*)-*threshold* is a method of sharing a* key K* among a set of *n* participants (denoted by *P*), in such a way that any *t* participants can compute the value of *K*, but no group of *t* − 1 participants can do so.

For the example of (2,3)-threshold, the value of *K* is chosen by a honest participant called the* dealer* (denoted by *D*, *D* ∉ *P*, where *P* is a set of participants). If *D* wants to share *K* among the participants in *P*, *D* distributes some partial information of *K* (called a* share*) for each participant. The shares should be distributed secretly, so no participant knows the share given to another participant. That is, an arbitrary participant does not know the information of *K* in (2,3)-threshold. In order to reconstruct a *K*, two or more participants should get together by an arbitrary algorithm.

In Shamir's scheme, the linear combination polynomial and Lagrange's interpolation arithmetic operations over prime *p* were used in order to distribute and reconstruct a *K*, respectively. It consists of three phases with (*t*, *n*)-threshold: initialization, share distribution, and reconstruction.

#### 2.1.1. Initialization Phase

*D* chooses *n* distinctly nonzero elements of *Z*_*p*_, denoted by *x*_*i*_, 1 ≤ *i* ≤ *n* (where *p* ≥ *n* + 1). For 1 ≤ *i* ≤ *n*, *D* gives the value *x*_*i*_ to *P*_*i*_. The value *x*_*i*_ is public.

#### 2.1.2. Share Distribution Phase

When *D* wants to share a key *K* ∈ *Z*_*p*_, *D* secretly chooses *t* − 1 elements of *Z*_*p*_ which are denoted as *a*_1_,…, *a*_*t*−1_. And then *D* computes *y*_*i*_ = *f*(*x*_*i*_), for 1 ≤ *i* ≤ *n*, by

(2) $$\begin{array}{c} y_{i}=f\left(x_{i}\right)=K+\overset{t-1}{\underset{j=1}{\sum }}a_{j}x_{i}^{j}\left(\mathrm{mod}p\right), \end{array}$$

where *a*_1_, *a*_2_,…, *a*_*t*−1_ are randomly determined from integers within [0, *p* − 1]. Finally, *D* distributes the share *y*_*i*_ to *P*_*i*_.

#### 2.1.3. Reconstruction Phase

If participants want to reconstruct a *K*, *t* or more participants will be recruited by *D*. *K* is reconstructed with information (*x*_*i*_, *f*(*x*_*i*_)) for each participant *P*_*i*_ and Lagrange interpolation formula as shown in (3) for polynomials.

Consider

(3) $$\begin{array}{c} f\left(x\right)=\overset{t}{\underset{j=1}{\sum }}\left(y_{i},\underset{1\leq k\leq t,k\neq j}{\prod }\frac{x-x_{i_{k}}}{x_{i_{j}}-x_{i_{k}}}\right)\mathrm{mod}p. \end{array}$$

Lastly, a key can be derived from *f*(0) = *K*.

### 2.2. Renvall and Ding's (*t* − 1,  *n*)-Threshold Nonlinear Secret Sharing

In 1996, Renvall and Ding [21] have proposed a nonlinear secret sharing scheme. A polynomial arithmetic technique is based on quadratic form (called a nonlinear combination) instead of the linear combination. In fact, it is an inner product for an arbitrary matrix, and detailed arithmetic is as follows [21].

Let *p* be a large prime of the form *p* ≡ 3 (mod⁡ 4). In order to generate the secret, it should be within [0, (*p* − 1)/2]. All arithmetic operations are performed over Galois field (GF(*p*)). For any positive integers *t*, *n* (*t* ≤ *n*) and each set of indices 1 ≤ *i*_1_ < ⋯<*i*_*t*_ ≤ *n*, Vandermonde matrix M is generated by

(4) $$\begin{array}{c} M=\left(\begin{array}{cccc} {\left(a_{i_{1}}\right)}^{0} & {\left(a_{i_{1}}\right)}^{1} & \cdots & {\left(a_{i_{1}}\right)}^{t-1}\\ {\left(a_{i_{2}}\right)}^{0} & {\left(a_{i_{2}}\right)}^{1} & \cdots & {\left(a_{i_{2}}\right)}^{t-1}\\ \vdots & \vdots & \ddots & \vdots \\ {\left(a_{i_{t}}\right)}^{0} & {\left(a_{i_{t}}\right)}^{1} & \cdots & {\left(a_{i_{t}}\right)}^{t-1} \end{array}\right), \end{array}$$

where all elements in matrix M are distinct nonzero over GF(*p*) and M must satisfy two requirements as follows.

R1: for any set of indices 1 ≤ *i*_1_ < ⋯<*i*_*t*−1_ ≤ *n*,
  (5) $$\begin{array}{c} 1+\overset{t-1}{\underset{u=1}{\sum }}{\left(\overset{t-2}{\underset{v=1}{\sum }}N\right)}^{2}\neq 0, \end{array}$$
 where $N=\left[n\;{\left(i_{1},i_{2},\ldots ,i_{t-1}\right)}_{u,v}\right]$ is the inverse of M by the Chinese remainder algorithm.
R2: for any set of indices 1 ≤ *i*_1_ < ⋯<*i*_*t*−2_ ≤ *n*, one of the following conditions is held:
  (1) *δ* ≠ 0 and *δ* ^2^ − 4*βγ* is a quadratic residue,
  (2) *δ* = 0, *γ* ≠ 0, and −*β*/*γ* is a quadratic residue,
 where *β*, *δ*, and *γ* are expressed by (6), (7), and (8), respectively.
 Consider
  (6) $$\begin{array}{c} \beta =1+\overset{t-1}{\underset{u=1}{\sum }}{\left(\overset{t-2}{\underset{v=1}{\sum }}n{\left(i_{1},i_{2},\ldots ,i_{t-1}\right)}_{u,v}\right)}^{2}, \end{array}$$
  (7) $$\begin{array}{c} \gamma =1+\overset{t-1}{\underset{u=1}{\sum }}{\left(\overset{t-2}{\underset{v=1}{\sum }}n{\left(i_{1},i_{2},\ldots ,i_{t-1}\right)}_{u,v}{\left(a_{i_{t}}\right)}^{t-1}\right)}^{2}, \end{array}$$
  (8) $$\begin{array}{c} \delta =2\overset{t-2}{\underset{u=1}{\sum }}\left(\overset{t-2}{\underset{v=1}{\sum }}N\right)\left(\overset{t-2}{\underset{v=1}{\sum }}N{\left(a_{i_{t}}\right)}^{t-1}\right), \end{array}$$
 where $N=[n{\left(i_{1},i_{2},\ldots ,i_{t-1}\right)}_{u,v}]$.

In sharing process, the secret *s*_1_ with 0 ≤ *s*_1_ ≤ (*p* − 1)/2 is selected to be shared among *n* participants. Then, *s*_2_,…, *s*_*t*_ are randomly chosen over GF(*p*). Let a set **s** = {*s*_1_, *s*_2_,…, *s*_*t*_}. Each share is calculated by

(9) $$\begin{array}{c} f\left(x\right)=xx^{T}={\left(x_{1}\right)}^{2}+{\left(x_{2}\right)}^{2}+\cdots +{\left(x_{t}\right)}^{2}\;\text{over}\;\text{GF}\left(p\right), \end{array}$$

where **x** is a row vector (or row matrix which has a single row of *t* elements) and it can be expressed as **x** = [*x*_1_ *x*_2_ ⋯ *x*_*t*_], and **x** ^*T*^ is a transpose of **x**.

In order to distribute the shares, *D* calculates *k*_0_ and *k*_*i*_*j*__ as follows:

(10) $$\begin{array}{c} k_{0}=f\left(s\right)={\left(s_{1}\right)}^{2}+\cdots +{\left(s_{t}\right)}^{2},\\ k_{i}=f\left(s+\alpha_{i_{j}}\right)={\left(s_{1}+{\left(a_{i_{1}}\right)}^{0}\right)}^{2}+\cdots +{\left(s_{t}+{\left(a_{i_{1}}\right)}^{t-1}\right)}^{2}, \end{array}$$

where *α*_*i*_*j*__ is *i*_*j*_th row vector in a Vandermonde matrix M (as shown in (4)) and 1 ≤ *i*_*j*_ ≤ *n*. Then *D* distributes (*k*_0_, *k*_*i*_*j*__) to participant *P*_*i*_*j*__.

If participants want to reconstruct the secret *s*, *t* − 1 or more participants will be recruited by *D*. And then, *s*_1_ is reconstructed with information (*k*_0_, *k*_*i*_*j*__) for each participant *P*_*i*_*j*__ and as follows:

(11) $$\begin{array}{c} k_{0}=f\left(s\right),\;\;k_{i_{j}}-k_{0}=2\alpha_{i_{j}}s^{T}+\alpha_{i_{j}}\alpha_{i_{j}}^{T}. \end{array}$$

They have completed verification of security for Tompa-Woll attack [21]. In this paper, we propose a nonlinear secret image sharing based on concepts of their scheme and steganography.

## 3. The Proposed Scheme

In this section, we illustrate considerations, sharing, and reconstruction algorithms.

### 3.1. Considerations

In order to propose a new nonlinear secret image sharing scheme, we discuss some considerations such as handing techniques of secret and shadow images and overflow (or underflow).

#### 3.1.1. Handling of Secret Image

In previous scheme, they used (*t* − 1, *n*)-threshold concept. But we adapt a concept of (*t*, *n*)-threshold because of the convenience of proposed scheme. Given (*t*, *n*)-threshold, we require that the secret image (SI) to be divided into *n* shadow images (SHIs), and SI cannot be reconstructed without *t* or more SHIs. However, secrets (*s*_1_, *s*_2_,…, *s*_*t*_) are generated from SI's pixel values. The major difference between proposed and Renvall and Ding's schemes is that random value does not use *s*_2_,…, *s*_*t*_. Also, the range of prime *p* should be at least 60 bits in their scheme, but we let the range be 2 ≤ *p* ≤ 251. Tompa-Woll attack can occur because the range of prime *p* is less than 60 bits. But, our scheme is safe for this attack because it is based on the steganography concept that the secret is hidden in friendly cover image such as Lena, airplane, and baboon.

#### 3.1.2. Overflow and Underflow

If an arbitrary pixel value in SI is one of 251 to 255, it can occur an overflow or underflow because all arithmetic operations are performed within GF(251). So, we define another variable positive integer *m* (2 ≤ *m* ≤ *p*) in order to prevent overflow or underflow. A pixel value in SI is converted by *m*-ary form. For example, if a pixel value and *m* are 160_(10)_ and 7, respectively, converted pixel value is 316_(7)_. This converting technique is to provide reinforcement of security robustness. The converted *m*-ary values utilize inputs of nonlinear polynomial arithmetic. And then, its outputs are performed with modulo-*p* operation. Even if an attacker knows sharing and reconstruction algorithm of the proposed scheme, it is difficult to extract the correct SI.

#### 3.1.3. The Generation of Shadow Image

In Renvall and Ding's scheme, (*k*_0_, *k*_*i*_*j*__) is just a shadow. It was distributed to the participant *P*_*i*_*j*__ by the dealer *D*. However, the shadow is an image that (*k*_0_, *k*_*i*_*j*__) is embedded in the proposed scheme. In order to generate the shadow image, hence, we utilize XOR Boolean algebra operation for *k*_0_ and *k*_*i*_*j*__.

### 3.2. Sharing Procedure

Suppose that the cover image (CI), shadow image (SHI), and secret image (SI) consist of *M* × *M*, *M* × *M* and *N* × *N* pixels, and these represent CI = {*C*_0_, *C*_1_,…, *C*_*M*^2^−1_}, SHI = {SH_0_, SH_1_,…, SH_*M*^2^−1_}, and SI = {*S*_0_, *S*_1_,…, *S*_*N*^2^−1_}, respectively. In order to determine the number of participants, the dealer (*D*) decides to fix the threshold values *t* and *n* (*t* ≤ *n*). Also, *D* securely chooses a prime *p* and positive integer *m* (*m* ≤ *p*).

In this procedure, the sharing process is described.

 Input: A CI with size of *M* × *M* and a SI with size of *N* × *N*.
 Output: *n* SHIs with size of *M* × *M*.

**Step 1 .**

Convert a *i*th pixel value (*S*_*i*_) in SI into *m*-ary's expression as follows:

(12) $$\begin{array}{c} S_{i}⟹\left\{s_{i\lceil \log_{m}255\rceil },\ldots ,s_{(i+1)\lceil \log_{m}255\rceil -1}\right\}, \end{array}$$

where 0 ≤ *i* ≤ *N* ^2^ − 1 and *m* ≤ *p*. For example, if *m* is 3, *s*_*i*_ is one of 0, 1, or 2. Let a set **S** consist of *r* subsets that compose *tm*-ary's values and it is expressed by

(13) $$\begin{array}{c} S=\left\{s^{0}=\left(s_{0},\ldots ,s_{t-1}\right),s^{1}=\left(s_{t},\ldots ,s_{2t-1}\right),\ldots ,s^{r-1}\right\}. \end{array}$$

If (*N* ^2^ − 1)⌈log⁡_*m*_255⌉ is not a multiple of *t*, the remaining part in the last subset *s* ^*r*−1^ is filled by using well-known padding techniques.

**Step 2 .**

Choose **α**_*j*_ = ((*a*_*j*_)^0^, (*a*_*j*_)^1^, (*a*_*j*_)^2^,…, (*a*_*j*_)^*t*−1^) for all participants by *D*, where *a*_*j*_ ∈ GF(*p*) and 1 ≤ *j* ≤ *n*. For any *t* participants, the matrix M which satisfies two requirements R1 and R2 is constructed as (14) in order to ensure the correctly selected **α**_*j*_. If M does not satisfy two requirements, *D* should select a new **α**_*j*_. Also, M will be used in the reconstruction procedure.

Consider

(14) $$\begin{array}{c} M=\left(\begin{array}{c} \alpha_{1}\\ \alpha_{2}\\ \vdots \\ \alpha_{t} \end{array}\right). \end{array}$$

**Step 3 .**

Calculate a shadow value *k*_*j*_^*l*^ = *f*(**s** ^*l*^ + **α**_*j*_) (where *k*_*j*_^0^ = *f*(**s** ^0^), 1 ≤ *j* ≤ *n* and 1 ≤ *l* ≤ *r* − 1) with *f*(**x**) (as shown in (9)) and **S** and **α**_*j*_ by *D*.

**Step 4 .**

Embed the generated *l*th shadow value (*k*_*j*_^0^ ⊕ *k*_*j*_^*l*^) for *j*-th participant into CI with LSB1 and LSB2 techniques by Table 1 in order to generate SHI_*j*_ (1 ≤ *l* ≤ *r* − 1 and 1 ≤ *j* ≤ *n*). “⊕” indicates XOR Boolean algebra operation. Table 1 shows the embedding method by prime *p*. For example, it corresponds to the range of 2^4^ < *p* ≤ 2^5^ when *p* is 19. Hence, *k*_*j*_^0^ ⊕ *k*_*j*_^*l*^ are embedded by LSB1 and LSB2 techniques for 3 pixels.

**Step 5 .**

Distribute the generated SHI_*j*_ into *j*th participant by *D*. And then, *D* stores *k*_*j*_^0^ (1 ≤ *j* ≤ *n*) for all *s* ^*l*^ (0 ≤ *l* ≤ *r* − 1).

### 3.3. Reconstruction Procedure

In this procedure, the reconstruction process is described.

 Input: *t*  SHI_*j*_s with size of *M* × *M*.
 Output: a reconstructed SI′ with size of *N* × *N*.

**Step 1 .**

Extract a *l*th shadow value (*k*_*j*_^0^ ⊕ *k*_*j*_^*l*^) from* j*th participant's SHI_*j*_ (1 ≤ *l* ≤ *r* − 1 and 1 ≤ *j* ≤ *k*), and *k*_*j*_^*l*^ by XOR Boolean operation (*k*_*j*_^0^ ⊕ *k*_*j*_^*l*^) ⊕ *k*_*j*_^0^.

**Step 2 .**

Calculate a *l*th converted *m*-ary's value **s** ^*l*^ (0 ≤ *l* ≤ *r* − 1) as follows:

(15) $$\begin{array}{c} k_{j}^{0}=f\left(s^{0}\right),\;\;k_{j}^{l}=f\left(s^{l}+\alpha_{j}\right),\\ k_{j}^{0}=f\left(s^{0}\right),\;\;k_{j}^{l}-k_{j}^{0}=2\alpha_{j}{\left(s^{l}\right)}^{T}+\alpha_{j}\alpha_{j}^{T}. \end{array}$$

**Step 3 .**

Convert the calculated *s*_0_, *s*_1_,…, *s*_(*N*^2^−1)⌈log⁡_*m*_255⌉_ into pixel values by *m*, and, reconstruct a SI′ with size of *N* × *N*.

## 4. Experimental Results

In this section, we analyze the security and efficiency of proposed scheme.

### 4.1. The Measurement Tools

In order to estimate the efficiency and security of secret image sharing schemes, there exist two typical measurement tools: the embedding capacity and PSNR. The embedding capacity means the amount of embedded secret data in a cover image, and it can evaluate the efficiency of secret image sharing technique. That is, if the embedding capacity of an arbitrary technique is more increased, we can say that this technique has a good efficiency. It is generally measured in bit-per-pixel (bpp) or bit.

PSNR is the abbreviation for “*peak signal-to-noise ratio*” and it is the ratio between the maximum possible power of a signal and the power of corrupting noise that affects the fidelity of its representation. Nowadays, PSNR is the most popular distortion measurement tool in the field of image and video coding and compression. It is usually measured in* decibels *(dB), and it is well known that these difference distortion metrics are not very well correlated with the human visible system (HVS). This might be a problem for their application in secret image sharing since sophisticated secret image sharing methods exploit in one way or the other effects of these schemes [22]. The detailed PSNR is represented by

(16) $$\begin{array}{c} \text{PSNR}=10\times \log\left(\frac{{\text{MAX}}^{2}}{\text{MSE}}\right), \end{array}$$

where MAX indicates the maximum possible pixel value of the image. It is 255 because greyscale test images were used in this paper. MSE is the abbreviation for “*mean squared error*” and it is represented by

(17) $$\begin{array}{c} \text{MSE}=\frac{1}{M^{2}}\overset{M^{2}-1}{\underset{i=0}{\sum }}{\left(C_{i}-\text{S}{\text{H}}_{i}\right)}^{2}, \end{array}$$

where *M* indicates the size of CI and SHI. *C*_*i*_ and SH_*i*_ are *i*th pixel values in CI and SHI, respectively. Given two greyscale images, if PSNR value is close to infinity (=∞), the distortion between two images is zero; that is, two images are the same. On the other hand, if PSNR value is close to zero, the distortion is higher; that is, two images are different. Generally, PSNR value is more than 35 dB, the difference between two images cannot be distinguished in HVS.

### 4.2. Analysis

**Figure 1:**
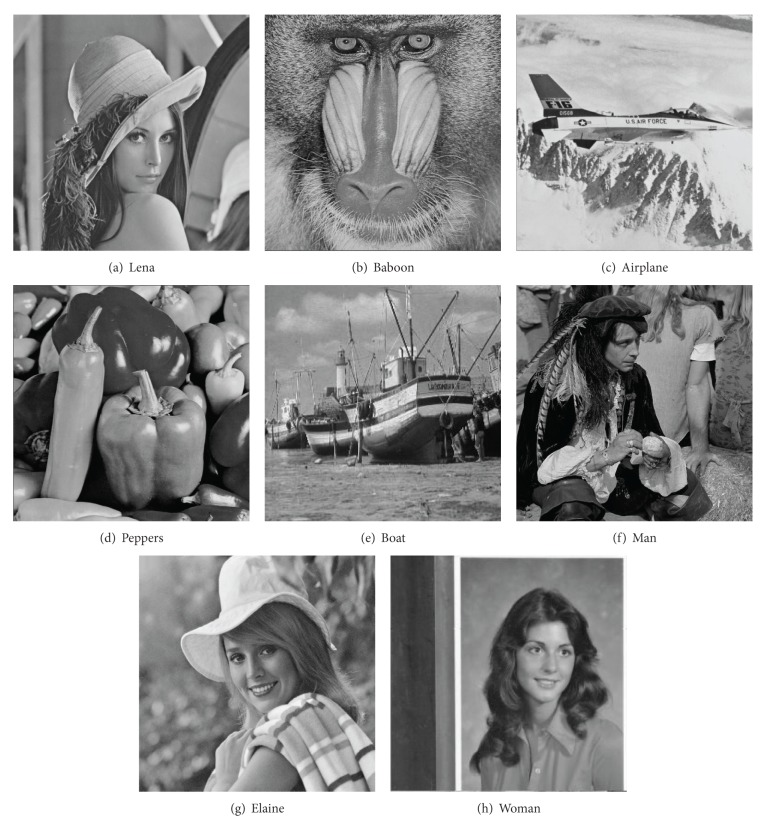
Eight greyscale test images.

In the experiments, we have performed the experiment for (3,4)-threshold and used eight greyscale test images as shown in Figure 1. The sizes of CI and SHI were 512 × 512 and 512 × 512, respectively. And the size of SI was 256 × 256 or 512 × 512, depending on the experiments. The secret data was generated by Rand function in C++ Library. And then, generated secret bitstream was composed by each eight-bit. In order to implement the proposed scheme, the OpenCV Library and C++ programming language (environment: MS Visual Studio 2010) were used.

In the proposed scheme, PSNR result depends on the embedding method by prime *p*'s interval as shown in Table 1. Our embedding method is that LSB1 and LSB2 were utilized. Hence, the minimum of PSNR without prime *p* is more than 44 dB because PSNR value of LSB2 embedding method is close to 44 dB in general. Depending on the change of prime *p*, PSNR result of the proposed scheme is shown in Table 2. If a prime *p* is 17, 19, or 29, PSNR values were higher than other values. This result was due to the embedding method in sharing phase. For example, maximum embedding capacity is 5-bit when the range of *p* is corresponding to 2^4^ < *p* ≤ 2^5^. And, 5-bit is embedded into 3 pixels in CI by LSB1 and LSB2 (i.e., LSB1 − LSB2 − LSB2). On the other hand, if *p* is 11, the number of maximum embedding bits is four and it is embedded into 2 pixels in CI by LSB2. So, PSNR result was less than that of 16 < *p* ≤ 32. Also, the number of maximum embedding bits is seven when *p* is 113. But PSNR values were less than that of range of 16 < *p* ≤ 32 because LSB2 embedding technique was utilized once more (i.e., LSB1 − LSB2 − LSB2 − LSB2). In the experimental result of PSNR, the minimum was 44.11 dB. This result shows that we cannot distinguish the distortion between CI and SHIs in HVS.

**Figure 2:**
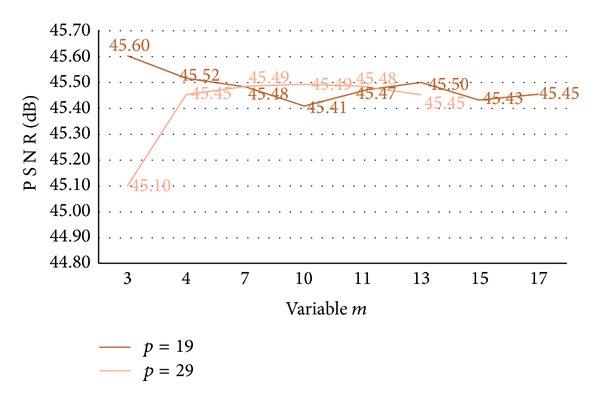
The relation of PSNR between *p* and *m*.

In the meantime, we utilized a variable *m* in order to prevent the overflow and reinforce the security for secret data. So, the variable *m* and prime *p* should not be correlated relatively and Figure 2 shows that the relation of PSNR between *p* and *m* with prime *p* as 19 and 29. PSNR values were located at 45.10 to 45.60 dB regardless of the increase (or decrease) of *m*. This result shows that there is no correlation between *p* and *m*.

The embedding capacity of the proposed scheme was decided with the embedding method by prime *p*'s interval as shown in Table 1. So, we have calculated the theoretical embedding capacity and it is shown in Table 3. MEBs, EC, *t*, and *M* indicate the number of maximum embedding bits, embedding capacity, threshold value, and size of shadow image, respectively. The embedding capacity is more increased when MEB is odd and *p* is close to 251. But, the embedding capacity is fixed at 2*tM* ^2^⌈log⁡_2_ *m*⌉ bit when MEB is even. This is because of the proposed embedding method as mentioned above. The best case is the intervals of 2^6^ < *p* ≤ 2^7^ and 2^7^ < *p* ≤ 2^8^ in terms of tradeoff between PSNR and the embedding capacity. And average of all embedding capacity values is 1.74*tM* ^2^⌈log⁡_2_ *m*⌉ bit.

In order to verify an excellence of the proposed scheme, we have performed a comparison between our and the previous schemes and the result of it is shown in Table 4. All results were average values and a unit of EC is bit-per-pixel (bpp). In EC results, the proposed and Lin and Chan's [18] schemes were related to a threshold value *t*. On the other hand, other schemes (Lin and Tsai [16], Wang and Shyu [23], and Chang et al. [24]) were fixed. This is because the amount of secret data that is inserted into polynomial is different. In typical schemes, the secret data is only embedded into the constant terms in the polynomial. But, the secret data is embedded into all coefficients except the highest order terms in Lin and Chan's scheme. If a threshold *t* is increased (this fact shows that the number of participants *n* is also increased), each EC is also increased more for our and Lin and Chan's schemes. But, EC and PSNR results in Lin and Chan's scheme have a inversely proportional relationship. In PSNR results, the proposed scheme was higher than others. We obtained that the efficiency and security of proposed scheme was superior to the previous schemes.

## 5. Conclusions

In this paper, we have proposed a (*t*, *n*)-threshold nonlinear secret image sharing scheme for the first time. Renvall and Ding's scheme was based on quadratic combination and Vandermonde matrix arithmetic operations. In the proposed scheme, it was extended to secret image sharing scheme with steganography concept. All arithmetic operations in the proposed scheme was limited within GF(251) because the target of secret was a pixel value in SI. In order to prevent the overflow in SHI and reinforce the security, a new variable *m* was used in sharing procedure. In sharing procedure, the embedding technique depended on LSB1, LSB2, and prime *p*. XOR Boolean algebra operation for embedding data was performed in order to increase PSNR and the embedding capacity. As the results, the average values of PSNR and the embedding capacity are 44.78 (dB) and 1.74*t*⌈log⁡_2_ *m*⌉ (bpp), respectively. Also, the best case of the proposed scheme is the intervals of 2^6^ < *p* ≤ 2^7^ and 2^7^ < *p* ≤ 2^8^ in terms of tradeoff between PSNR and the embedding capacity.

The future works are as follows: the studies of nonlinear secret image sharing scheme over GF(2^*n*^), the various experiments for PSNR and the embedding capacity, and the improved embedding technique.

## Figures and Tables

**Table 1:** The embedding method by prime *p*.

Range	Embedding technique	Pixel block
2^0^ < *p* ≤ 2^1^	LSB1	per 1 pixel
2^1^ < *p* ≤ 2^2^	LSB2	per 1 pixel
2^2^ < *p* ≤ 2^3^	LSB1 and LSB2	per 2 pixels
2^3^ < *p* ≤ 2^4^	LSB2	per 2 pixels
2^4^ < *p* ≤ 2^5^	LSB1 and LSB2	per 3 pixels
2^5^ < *p* ≤ 2^6^	LSB2	per 3 pixels
2^6^ < *p* ≤ 2^7^	LSB1 and LSB2	per 4 pixels
2^7^ < *p* ≤ 2^8^	LSB2	per 4 pixels

**Table 2:** PSNR result of (3,4)-threshold proposed scheme by prime *p*.

*p*	SHI_1_	SHI_2_	SHI_3_	SHI_4_	Average
11	44.08	43.92	43.93	43.96	43.97
17	45.39	45.47	45.60	45.39	45.46
19	45.51	45.48	45.50	45.44	45.48
29	45.49	45.38	45.43	45.35	45.41
37	44.15	44.13	44.13	44.04	44.11
79	45.18	45.17	45.03	45.16	45.13
113	45.08	45.11	45.12	45.10	45.10
167	44.15	44.13	44.20	44.15	44.16
251	44.16	44.13	44.16	44.20	44.16

**Table 3:** The theoretical embedding capacity of the proposed scheme.

Interval	MEBs	EC (bit)
2^0^ < *p* ≤ 2^1^	1	*tM* ^2^⌈log_2_⁡*m*⌉
2^1^ < *p* ≤ 2^2^	2	2*tM* ^2^⌈log_2_⁡*m*⌉
2^2^ < *p* ≤ 2^3^	3	$\frac{3}{2}tM^{2}\left\lceil \log_{2}m\right\rceil$
2^3^ < *p* ≤ 2^4^	4	2*tM* ^2^⌈log_2_⁡*m*⌉
2^4^ < *p* ≤ 2^5^	5	$\frac{5}{3}tM^{2}\left\lceil \log_{2}m\right\rceil$
2^5^ < *p* ≤ 2^6^	6	2*tM* ^2^⌈log_2_⁡*m*⌉
2^6^ < *p* ≤ 2^7^	7	$\frac{7}{4}tM^{2}\left\lceil \log_{2}m\right\rceil$
2^7^ < *p* ≤ 2^8^	8	2*tM* ^2^⌈log_2_⁡*m*⌉

**Table 4:** The comparison result between the proposed and previous schemes for the embedding capacity and PSNR.

Schemes	EC (bpp)	PSNR (dB)
Lin and Tsai [6]	1/2	39.17
Wang and Shyu [7]	1/4	—
Chang et al. [8]	3/4	40.92
Lin and Chan [22]	(*t* − 1)/3	40.01
Proposed	1.74*t*⌈log_2_⁡*m* ⌉	44.78
